# The prognostic role of inhalation injury in burn patients assessed by flexible bronchoscopy: a systematic review and meta-analysis

**DOI:** 10.3389/fmed.2026.1808915

**Published:** 2026-05-28

**Authors:** Erika Virdis, Giorgia Fara, Panagiotis Paliogiannis, Angelo Zinellu, Stefano Zoroddu, Arduino A. Mangoni, Biagio Di Lorenzo, Pietro Pirina, Alessandro G. Fois

**Affiliations:** 1 Department of Medicine, Surgery and Pharmacy, University of Sassari, Sassari, Italy; 2 Burn Center, University Hospital (AOU) of Sassari, Sassari, Italy; 3 Department of Biomedical Sciences, University of Sassari, Sassari, Italy; 4 Discipline of Clinical Pharmacology, College of Medicine and Public Health, Flinders University, Adelaide, SA, Australia; 5 Department of Clinical Pharmacology, Flinders Medical Centre, Southern Adelaide Local Health Network, Adelaide, SA, Australia; 6 Department of Medicine and Surgery, LUM University, Casamassima, Italy; 7 Clinical and Interventional Pneumology, University Hospital (AOU) of Sassari, Sassari, Italy

**Keywords:** airways, bronchoscopy, burns, inhalation injury, lung, smoke

## Abstract

**Introduction:**

Burn-related inhalation injury may follow an unpredictable clinical course, requiring careful post-exposure monitoring. Accordingly, there is a need for an assessment tool that not only accurately characterizes the extent of airway damage but also provides reliable prognostic information. Flexible bronchoscopy is currently considered the gold standard for evaluating the severity of inhalation injury; however, the prognostic value of bronchoscopic findings in burn patients remains controversial and not fully established.

**Methods:**

In this systematic review and meta-analysis, we aimed to evaluate the association between the bronchoscopically assessed severity of inhalation injury and key clinical outcomes, including mortality, hospital length of stay (HLS), intensive care unit length of stay (ICULS), duration of mechanical ventilation (VD), and the incidence of pneumonia.

**Results:**

The meta-analysis demonstrated significantly higher odds of mortality in patients with severe inhalation injury compared with those with mild injury (OR = 2.83, 95% CI: 1.79–4.48, *p* < 0.001). Statistically significant differences were also observed in HLS (pooled SMD = 0.93, 95% CI: 0.30–1.47, *p* = 0.002), ICULS (SMD = 0.53, 95% CI: 0.07–0.98, *p* = 0.02), and VD (SMD = 0.62, 95% CI: 0.19–1.06, *p* = 0.005), while the difference in pneumonia incidence between the high-grade and low-grade injury groups was not statistically significant (OR = 1.60, 95% CI: 0.63–4.11, *p* = 0.33).

**Conclusion:**

The results of the meta-analysis indicate strong associations between the severity of inhalation injury, as assessed by bronchoscopy, and several key prognostic indicators in burn patients with respiratory involvement. This suggests that early bronchoscopic assessment in these patients may provide valuable information for clinical management and improved care.

**Systematic review registration:**

https://www.crd.york.ac.uk/prospero/, identifier CRD42024518244.

## Introduction

Burn-related inhalation injury is a broad and nonspecific term encompassing injury to the airways and lung parenchyma resulting from thermal insult, chemical irritation, and systemic toxicity, predominantly caused by carbon monoxide (CO), cyanide, or their combined effects (1, 2). This condition is identified in up to 30% of patients requiring hospital admission following burn exposure and may involve isolated airway injury or progress to systemic toxicity (3, 4). Direct toxic injury is primarily mediated by low–molecular weight constituents of smoke, which, owing to their acidic or alkaline properties, propensity for free radical formation, and capacity to penetrate the distal airways and alveoli, cause extensive pulmonary damage (5). The resulting impairment in gas exchange and mucociliary clearance increases the risk of pneumonia, acute respiratory distress syndrome (ARDS), and mortality (6). Moreover, inhalation injury is a progressive process, with increasing severity closely associated with poorer clinical outcomes (7, 8). Accordingly, accurate assessment of inhalation injury severity may provide valuable prognostic information.

Bronchoscopy is widely considered the gold standard for assessing the severity and extent of airway and pulmonary injury following inhalation exposure (3, 9–11). In addition to enabling direct visualization of the airway mucosa, bronchoscopy facilitates the collection of respiratory specimens for microbiological analysis, supports targeted antimicrobial therapy, and allows therapeutic bronchial toilet, thereby improving ventilation and reducing the risk of atelectasis and pneumonia (10–13). Despite these advantages, there is no universal consensus regarding the prognostic value of bronchoscopy in inhalation injury (14). A primary source of disagreement stems from the operator-dependent nature of the procedure, as injury assessment may be influenced by both the clinician’s level of experience and the inherent subjectivity associated with endoscopic image interpretation.

To address these limitations and enhance interobserver consistency, several bronchoscopic scoring systems have been developed over time. These scoring systems are based on endoscopic findings and aim to standardize the assessment of inhalation injury severity while providing prognostic information related to patient outcomes (4). Among the proposed tools, the three most commonly used are the Abbreviated Injury Score (AIS) (9), the Inhalation Injury Severity Score (I-ISS) (15), and the Bronchoscopic Mucosal Score (MS) (16). Each scoring system employs an ordinal scale (AIS: 0–4; I-ISS and MS: 0–3), with higher scores indicating greater injury severity (9, 15, 16). Despite differences in scale structure and nomenclature, all systems are based on the bronchoscopic assessment of key mucosal features, including the presence or absence of erythema, edema, and necrosis.

In a recent study, Flinn et al. (4) compared several bronchoscopic assessment scales to determine which provided the greatest prognostic value. The three most used scoring systems (AIS, I-ISS, and MS) were found to demonstrate comparable prognostic performance. Numerous clinical studies have subsequently explored the relationship between inhalation injury severity and clinical outcomes in an effort to clarify the prognostic significance of bronchoscopic evaluation (2–4, 9, 15–26).

In this context, the objective of this systematic review and meta-analysis was to evaluate the available evidence on the severity of burn-related inhalation injury as assessed by bronchoscopy, and its association with key clinical outcomes, including mortality, hospital length of stay (HLS), intensive care unit length of stay (ICULS), duration of mechanical ventilation (VD), and the incidence of pneumonia.

## Materials and methods

### Search strategy, eligibility criteria, and study selection

PubMed and Scopus databases were systematically searched from inception to 30 November 2024 using the following search strategy: “Inhalation Injury” OR “inhalation injury” OR “inhalation injuries” OR “thermal airway injury” OR “thermal airway injuries” OR “inhalation burn” OR “inhalation burns” OR “smoke inhalation” OR “smoke inhalation injury” AND “Bronchoscopy” OR bronchoscopy OR bronchoscopic OR “airway endoscopy” OR “respiratory endoscopy” OR “fiberoptic bronchoscopy” OR “fibreoptic bronchoscopy.” Only studies that included endoscopic evaluation for assessing the severity of inhalation injury in adults were included. Studies were excluded if they focused on non-thermal burns (e.g., chemical or electrical injuries), involved pediatric populations, did not assess the prognostic value of bronchoscopy, or were not published in English. Reference lists were screened to identify additional records.

Patients were stratified into two broad categories: “low-grade injury” and “high-grade injury.” The low-grade group comprised patients showing no or only mild signs of erythema and edema, without necrosis or airway blockage. This group corresponds to grades 0, 1, and 2 on the AIS scale, and grades 0 and 1 on both the I-IIS and MS scales. The high-grade group included patients with more severe findings such as erythema, edema, mucosal necrosis, or carbonaceous deposits. This group corresponds to grades 3 and 4 on the AIS scale, and grades 2 and 3 on the I-IIS and MS scales.

The study was carried out in accordance with the PRISMA 2020 guidelines for reporting systematic reviews and meta-analyses (27). The quality, relevance, and potential bias of the included studies were evaluated using the Joanna Briggs Institute (JBI) Critical Appraisal Checklist for analytical studies (28). To assess the certainty of the evidence, the GRADE (Grading of Recommendations, Assessment, Development and Evaluation) framework was applied (29). The review protocol was registered in the International Prospective Register of Systematic Reviews (CRD42024518244).

### Statistical analysis

Differences in mortality and pneumonia incidence between patients with high- and low-grade inhalation injuries were analyzed using odds ratios (ORs) with 95% confidence intervals (CIs). Variations in HLS, ICULS, and VD were assessed using standardized mean differences (SMDs) with 95% CIs. When required, medians with interquartile ranges or minimum-maximum values were converted to means and standard deviations using the method proposed by Wan et al. (30). All outcomes were summarized in forest plots for continuous data, with statistical significance set at *p* < 0.05.

Heterogeneity and inconsistency across studies were assessed using the Q statistics and the I^2^ index, respectively (31, 32). A random-effects model was employed when I^2^ was 50% or higher. To evaluate the robustness of the overall risk estimate, sensitivity analyses were conducted by examining the influence of individual studies (33). Potential publication bias was assessed using Begg’s rank correlation test (34), Egger’s regression asymmetry test (35), and the “trim-and-fill” method (36). Where possible, univariate meta-regression analyses were performed to explore associations between outcomes and study design, publication year, gender, and total body surface area. All statistical analyses were carried out using Stata version 14 (StataCorp, College Station, TX, United States).

## Results

### Systematic search

**FIGURE 1:**
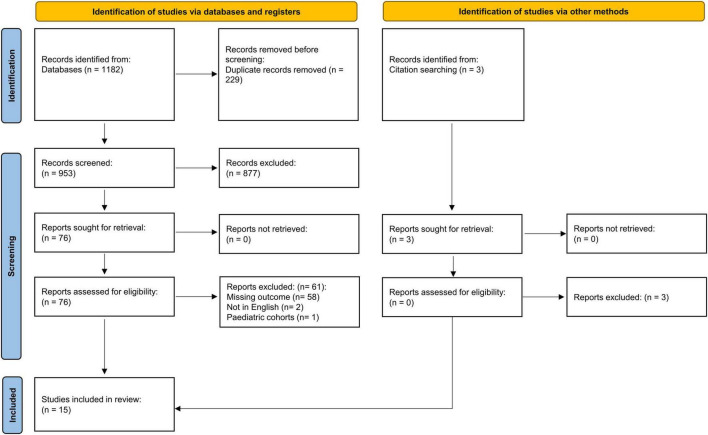
Flow-chart illustrating the study selection process.

A total of 1,182 records were identified through database searching and three records through initial reference screening (Figure 1). Following duplicate removal and relevance screening, 76 full-text articles were assessed for eligibility, of which 15 met the inclusion criteria (2, 3, 9, 15, 17–27). The remaining 61 articles were excluded due to lack of relevant outcomes (*n* = 58), non-English language (*n* = 2), or inclusion of pediatric populations (*n* = 1). No additional studies were identified through manual reference screening of eligible studies; however, some relevant studies may have been missed due to the limited number of databases searched.

Risk of bias was rated as low in ten studies (2, 3, 18, 21–27), and moderate in the remaining five (9, 15, 17, 19, 20) (Supplementary Table 1). The overall certainty of evidence was initially rated as low due to the case-control design of the included studies (rating 2).

### Burn—related inhalation injury and mortality

**FIGURE 2:**
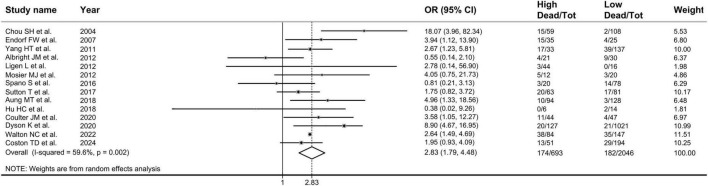
Forest plot of burn—related inhalation injury severity and mortality.

Fourteen studies examined the impact of exposure on mortality (3, 9, 15, 17–19, 21–27), comparing high-grade versus low-grade inhalation injuries across a total of 2,739 patients (Tables 1, 2). Six studies were conducted in the USA (3, 9, 18, 22, 27), three in China (15, 19, 24), two in Canada (21, 23), and one each in Korea (17), Australia (26), and the UK (2). Most studies (*n* = 12) had a retrospective study design (2, 3, 9, 17, 19, 21–27), while two were prospective (15, 18). Mortality data synthesis is presented in Figure 2. Due to substantial heterogeneity among studies (I^2^ = 59.6%, *p* = 0.002), a random-effects model was used. The pooled odds ratio (OR) showed a significantly increased risk of mortality in patients with high-grade injuries compared to those with low-grade injuries (OR = 2.83, 95% CI: 1.79–4.48, *p* < 0.001). Sensitivity analysis revealed that the OR ranged from 1.90 to 2.47. Both the Begg’s (*p* = 0.16) and Egger’s tests did not suggest the presence of bias (*p* = 0.64); also, the “trim-and-fill” method did not identify any missing studies for inclusion in the funnel plot.

**TABLE 1:** Characteristics of the studies and clinical outcomes of patients with low grade burn—related inhalation injury.

Study name, country, year	Type	Low grade inhalation injury							
		n	Males (%)	TBSA (%)	Dead (n)	HLS (mean ± sd)	ICULS (mean ± sd)	VD (mean ± sd)	Pn (n)
Chou SH et al. China, 2004	P	108	–	–	2	–	–	–	–
Endorf FW et al. USA, 2007	R	25	–	6.6	4	–	–	8.6 ± 1.4	–
Yang HT et al. Korea, 2011	R	137	–	45.4	39	–	–	–	–
Albright JM et al. USA, 2012	P	30	63	24.7	9	19.3 ± 27.2	18.7 ± 24.1	13.7 ± 21.8	13
Ligen L et al. China, 2012	R	16	–	–	0	7 ± 2	–	0 ± 0	0
Mosier MJ et al. USA, 2012	R	20	70	–	3	–	37.5 ± 22.9	23.8 ± 22.8	–
Bai C et al. China, 2013	R	12	–	–	–	4.2 ± 1.3	–	–	–
Spano S et al. Canada, 2016	R	78	–	–	14	–	–	17.2 ± 13.6	–
Sutton T et al. USA, 2017	R	81	69	19.3	17	17.8 ± 17.7	–	–	30
Aung MT et al. Canada, 2018	R	128	–	12	3		4.6 ± 3.5	1.7 ± 2.2	–
Hu HC et al. China, 2018	R	14	50	60	2	71.9 ± 35.2	57.7 ± 3.08	18.1 ± 9.6	–
Coulter JM et al. USA, 2020	R	47	100	36.5	4	–	–	–	–
Dyson K et al. Australia, 2020	R	1,021	75	–	21	9 ± 9.7	3.9 ± 4.2	48.7 ± 63.8	–
Walton NC et al. UK, 2022	R	147	–	–	35	31 ± 31.4	8 ± 12.7	10.1 ± 17.4	75
Coston TD et al. USA, 2024	R	194	71	13.8	29	20.6 ± 25.2	–	–	–

HLS, hospital length of stay; ICULS, intensive care unit length of stay; P, prospective; Pn, pneumonia; R, retrospective; TBSA, total burn surface area; VD, duration of ventilatory support.

**TABLE 2:** Characteristics of the studies and clinical outcomes of patients with high grade burn—related inhalation injury.

Study name, country, year	Type	High grade inhalation injury							
		n	Males (%)	TBSA (%)	Dead (n)	HLS (mean ± sd)	ICULS (mean ± sd)	VD (mean ± sd)	Pn (n)
Chou SH et al. China, 2004	P	59	–	–	15	–	–	–	–
Endorf FW et al. USA, 2007	R	35	–	6.7	15	–	–	12.8 ± 2.2	–
Yang HT et al. Korea, 2011	R	33	–	40	17	–	–	–	–
Albright JM et al. USA, 2012	P	21	0.52	14.7	4	27.7 ± 15.9	26.3 ± 18.3	23.7 ± 17.5	14
Ligen L et al. China, 2012	R	44	–	–	3	19.3 ± 3.5	–	5 ± 2	9
Mosier MJ et al. USA, 2012	R	12	0.67	–	5	–	57.6 ± 21.3	30.6 ± 7.8	–
Bai C et al. China, 2013	R	8	–	–	–	15.8 ± 4.2	–	–	–
Spano S et al. Canada, 2016	R	20	–	–	3	–	–	23.2 ± 16.4	–
Sutton T et al. USA, 2017	R	63	0.65	20.7	20	28.7 ± 24.3	–	–	34
Aung MT et al. Canada, 2018	R	94	–	18	10	–	152.2 ± 159.8	112 ± 144.5	–
Hu HC et al. China, 2018	R	6	0.67	45.5	0	62 ± 23.8	43.7 ± 24.9	11.8 ± 6.5	–
Coulter JM et al. USA, 2020	R	44	1.00	49.1	11	–	–	–	–
Dyson K et al. Australia, 2020	R	127	0.76	–	20	16 ± 15	6.3 ± 6.5	103 ± 126.7	–
Walton NC et al. UK, 2022	R	84	–	–	38	26 ± 35.5	13.7 ± 20.4	9.8 ± 15.5	50
Coston TD et al. USA, 2024	R	51	–	13.2	13	35 ± 39.7	–	–	–

HLS, hospital length of stay; ICULS, intensive care unit length of stay; P, prospective; Pn, pneumonia; R, retrospective; TBSA, total burn surface area; VD, duration of ventilatory support.

The univariate meta-regression analyses found no significant relationships between effect size and study design (*t* = 1.57; *p* = 0.14), year of publication (*t* = –1.32; *p* = 0.21), gender (*t* = –0.31; *p* = 0.76), or total burn surface area (TBSA) (*t* = –0.26; *p* = 0.80). The subgroup analyses also revealed no significant differences based on study design (retrospective studies: OR = 2.27, 95% CI 1.58–3.25, *p* < 0.001, *I*^2^ = 59.6%, *p* = 0.04; prospective studies: OR = 2.84, 95% CI 0.12–64.8, *p* = 0.52, *I*^2^ = 92%, *p* = 0.001) or geographic areas (Asia: OR = 2.78, 95% CI 0.66–11.64, *p* = 0.16, *I*^2^ = 68.4%, *p* = 0.02; North America: OR = 1.75, 95% CI 1.20–2.55, *p* = 0.01, *I*^2^ = 29.4%, *p* = 0.19). However, notable reductions in heterogeneity were observed among retrospective studies (*I*^2^ = 59.6%, *p* = 0.004) and studies conducted in North America (*I*^2^ = 29.4%, *p* = 0.19).

The overall certainty of evidence remained low (rating 2) after taking into account the low to moderate risk of bias across all studies (no change), the substantial but partly explainable heterogeneity (no change), the absence of indirectness (no change), the moderate effect size (OR = 1.90, no change), and the lack of publication bias as indicated by Begg’s test, though not Egger’s test; additionally, the “trim-and-fill” method did not identify any missing studies to add to the funnel plot (no change).

### Burn—related inhalation injury and hospital length of stay (HLS)

**FIGURE 3:**
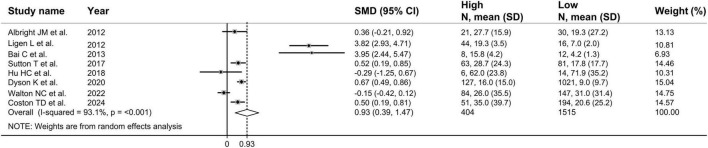
Forest plot of burn—related inhalation injury severity and hospital length of stay.

Eight studies investigated HLS as an outcome (2, 18, 19, 20, 22, 24, 27), encompassing a total of 1,919 participants. These studies were conducted in China [three studies (19, 20, 24)], the USA [three studies (18, 22, 27)], Australia [one study (26)], and the UK [one study (2)]. Seven studies had a retrospective design (2, 19, 20, 22, 24, 26, 27), while only one was prospective (18). Due to high heterogeneity (*I*^2^ = 93.1%, *p* < 0.001), a random-effects model was applied to synthesize the data, as illustrated in the forest plot of HLS duration (Figure 3). The pooled standardized mean difference (SMD) showed a significant increase in hospital stay for patients with high-grade injury compared to low-grade (SMD = 0.93, 95% CI 0.39–1.47, *p* < 0.001). Sensitivity analysis, conducted by removing each study individually, produced SMD estimates ranging from 0.53 to 1.31. Due to the limited number of studies, tests for publication bias, meta-regression, and subgroup analyses were not performed. The overall certainty of evidence was downgraded to very low (rating 1) due to the high, unexplained heterogeneity and the absence of publication bias assessment.

### Burn—related inhalation injury and intensive care unit length of stay (ICULS)

**FIGURE 4:**
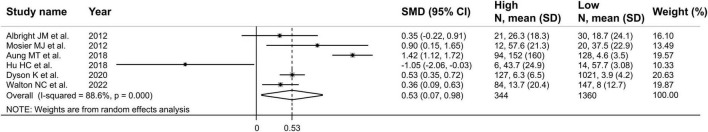
Forest plot of burn—related inhalation injury severity and intensive care unit length of stay.

Six studies (2, 3, 18, 23, 24, 26) involving 1,704 patients assessed ICULS. Two were conducted in the USA (3, 18), and one each in Canada (23), China (24), Australia (26), and the UK (2). Five studies had a retrospective design (2, 3, 23, 24, 26), while one was prospective (18). Given the high heterogeneity among studies (*I*^2^ = 88.6%, *p* < 0.001), a random-effects model was applied for data synthesis (Figure 4). The pooled standardized mean difference (SMD) showed a significant difference in ICULS between high- and low-grade injury groups (SMD = 0.53, 95% CI 0.07–0.98, *p* = 0.02). Sensitivity analysis indicated that no single study substantially influenced the result, with SMD estimates ranging from 0.37 to 0.71. Due to the limited number of studies, tests for publication bias, meta-regression, and subgroup analyses were not performed. The overall certainty of evidence was downgraded to very low (rating 1) because of the high unexplained heterogeneity and the absence of publication bias assessment.

### Burn—related inhalation injury and duration of ventilatory treatment (VD)

The VD was assessed in nine studies (2, 3, 9, 18, 19, 21, 23, 24, 26) including 1,922 patients. Three studies were conducted in the USA (3, 9, 18), two each in Canada (21, 23) and China (19, 24), and one each in Australia (26) and the UK (2). Only the study by Albright et al. had a prospective design (18), while the remaining eight studies were retrospective (2, 3, 9, 19, 21, 23, 24, 26).

**FIGURE 5:**
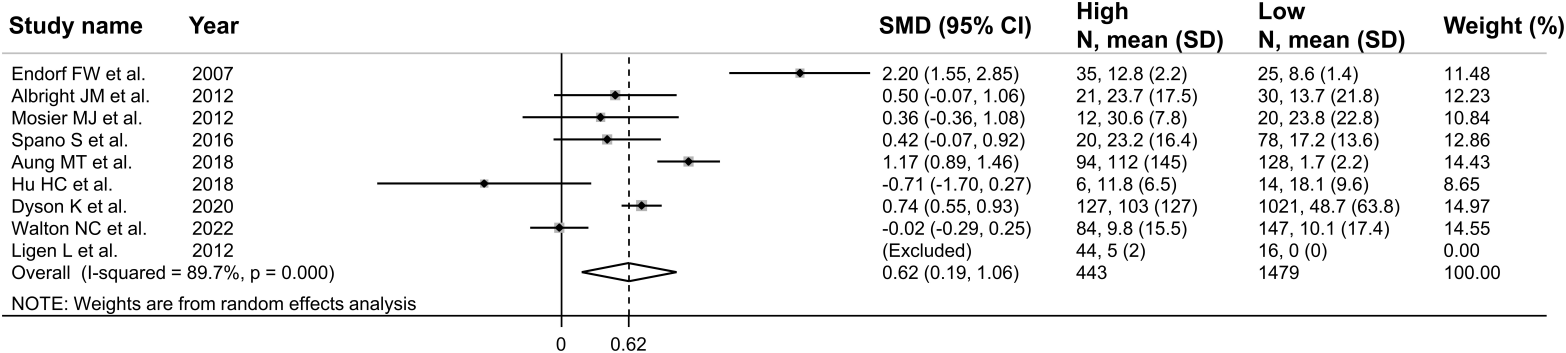
Forest plot of burn—related inhalation injury severity and ventilatory support duration.

Due to substantial heterogeneity across studies (*I*^2^ = 89.7%, *p* < 0.001), a random-effects model was used. The pooled standardized mean difference (SMD) showed a significant difference in VD between high- and low-grade injury groups (SMD = 0.62, 95% CI 0.19–1.06, *p* = 0.005), as illustrated in the forest plot (Figure 5). Because of the limited number of studies, publication bias tests, meta-regression, and subgroup analyses were not conducted. The overall certainty of evidence was downgraded to very low (rating 1) due to the high unexplained heterogeneity and the lack of publication bias assessment.

### Pneumonia development

**FIGURE 6:**
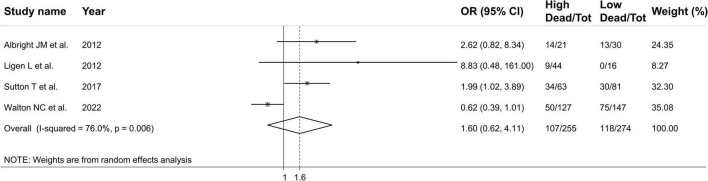
Forest plot of burn—related inhalation injury severity and pneumonia development.

Pneumonia development was reported as an outcome in four studies (2, 18, 19, 22), including a total of 486 participants. Two studies were conducted in the USA (18, 22), one in China (19), and one in the UK (2). Due to the limited number of studies, only an odds ratio (OR) for pneumonia development was calculated and presented in a forest plot (Figure 6). The studies exhibited substantial heterogeneity (*I*^2^ = 76.0%, *p* = 0.006), and no significant difference was found between high- and low-grade injury groups (pooled OR = 1.60, 95% CI 0.63–4.11, *p* = 0.33). The overall certainty of evidence was downgraded to very low (rating 1) because of the high unexplained heterogeneity and the absence of publication bias assessment.

## Discussion

The results of the meta-analysis indicate strong associations between the severity of inhalation injury, as assessed by bronchoscopy, and several key prognostic indicators in burn patients with respiratory involvement. Specifically, the differences in clinical outcomes and mortality between patients with high-grade airway damage and those with low-grade airway injury, suggests the clinical utility of flexible bronchoscopy as a tool for evaluating burn-related inhalation injury and, consequently, its value in guiding appropriate clinical decision-making in burn patients.

Patients with high grade injury show moderate to severe degrees of erythema, mucosal edema, ulcerations and necrosis while patients with low-grade injury are characterized by absence or presence of mild mucosal erythema and edema, and this can be easily determined via flexible bronchoscopy in the early phases after burn trauma. This meta-analysis showed a significant increase in the mortality odds for patients with high grade injury compared to the low-grade group, irrespective of the design of the studies included.

This difference may be explained by the pathophysiology of airway and pulmonary injury. Many combustion products act as asphyxiants by reducing oxygen availability and impairing cellular respiration, either by displacing oxygen at the alveolar level or by interfering with oxygen transport and utilization through hemoglobin binding (as in CO) and inhibition of cytochrome oxidase (as in CO and hydrogen cyanide) (37). In addition, exposure to smoke and toxic compounds triggers the release of reactive oxygen species (ROS), which interact with endothelial nitric oxide, leading to upper airway edema, reduced protein permeability, increased permeability to small solutes, elevated pulmonary microvascular pressure, and loss of hypoxic pulmonary vasoconstriction (38, 39). These processes result in pulmonary edema, decreased lung compliance due to increased extravascular lung fluid and pulmonary lymph flow, and rapid inactivation of surfactant (38, 39). The resulting impairment in gas exchange and airway clearance, together with ventilation—perfusion mismatch, substantially increases the risk of ARDS and mortality (6). These effects may be exacerbated by subglottic stenosis and airway obstruction, leading to progressively worsening systemic consequences, including reduced hemoglobin levels and oxygen delivery, systemic release of pro-inflammatory mediators, immunosuppression, pneumonia, and other complications, ultimately increasing overall morbidity and mortality (1).

In the meta-regression analysis, effect size was not significantly associated with year of publication, sex or TBSA. Notably, a significant difference in effect size was observed between studies conducted in Asia and those conducted in North America, suggesting that ethnicity may play a role in modulating the association between inhalation injury and mortality. Although the primary analysis demonstrated substantial heterogeneity across studies, subgroup analyses identified markedly reduced heterogeneity, particularly among North American studies (*I*^2^ = 39.5%) and in retrospective studies (*I*^2^ = 48.5%). Nevertheless, it should be kept in mind that residual confounding is highly likely, as high-grade inhalation injury often coexists with larger TBSA burns, enclosed-space fires, longer exposure times, and greater overall physiological insult. Therefore, the observed increase in mortality may reflect the overall severity of injury rather than the bronchoscopic grade of inhalation injury alone. Meta-regression for TBSA, however, was limited by the small number of available studies and inconsistent reporting and therefore cannot fully exclude this type of confounding.

In addition, when compared with the low-grade group, the high-grade group demonstrated statistically significant differences in HLS, ICULS, and VD. However, because of the limited number of available studies, assessments of publication bias, meta-regression, and subgroup analyses could not be performed, resulting in a relatively low overall level of certainty. These outcomes were characterized by a high degree of heterogeneity. This may again be related to the overall severity of the burn injury, as noted above, including the TBSA and the type and duration of thermal exposure, as well as other clinical factors such as the general condition of the patient, ventilation practices, intubation thresholds, and burn center variability. In addition, several technical factors, such as the timing of bronchoscopy, differences in overall clinical management, and regional variations in practice, may represent further sources of heterogeneity as they are difficult to identify, standardize, and account for in meta-analyses. For example, the timing of the first bronchoscopic evaluation, although crucial for assessing thermal airway injury, is not yet widely standardized and depends on several factors, including the overall severity of the injury and the patient’s clinical condition, the availability of endoscopic resources, local protocols, and others. Inhalation injury is a dynamic and progressive process that can evolve rapidly within the first 24–48 hours; therefore, early bronchoscopic assessment may be valuable for its detection, and repeated evaluations may be required for adequate follow-up (7, 8).

In contrast, with respect to the outcome of pneumonia development, the analysis was limited in a small number of studies with high heterogeneity and did not show any statistically significant difference between the low-grade and high-grade groups. Beyond these technical considerations, a lack of correlation between the severity of inhalation injury and the development of pneumonia has already been reported in previous studies, such as that by Flinn et al. (4). In this study, the authors suggested that the development of pneumonia in burns is driven by dynamic pathophysiological processes that cannot be captured at a single time point but rather should be assessed longitudinally as they evolve over time.

This meta-analysis has several important limitations, primarily related to the small number of available studies and their methodological constraints, particularly the limited number of prospectively designed trials. In addition, the high heterogeneity observed across studies for certain outcomes, the confounding factors previously discussed, and limitations arising from the difficulty in standardizing bronchoscopic scoring systems represent further issues. Nonetheless, the study also has notable strengths, including a rigorous assessment of risk of bias and certainty of evidence, as well as the absence of comparable meta-analyses in the existing literature. In any case, the need for further well-designed prospective studies in this setting needs to be underlined, like the multicenter prospective cohort study projected by the American Burn Association, with the aim of developing a scoring system for inhalation injury based on several combined clinical, radiographic, bronchoscopic, and biochemical parameters (40).

## Conclusion

The results of the meta-analysis indicate strong associations between the severity of inhalation injury, as assessed by bronchoscopy, and several key prognostic indicators in burn patients with respiratory involvement. Nevertheless, several methodological limitations that may affect the strength of these inferences have been identified, including difficulties in harmonizing bronchoscopy scoring systems, substantial heterogeneity, and a limited number of prospectively designed studies, among others. Despite these limitations, our findings overall suggest that early bronchoscopic assessment in these patients may provide valuable information for clinical management and improved care.

## Data Availability

The original contributions presented in the study are included in this article/Supplementary material, further inquiries can be directed to the corresponding author.
