# Supplementary material for: The prognostic role of inhalation injury in burn patients assessed by flexible bronchoscopy: a systematic review and meta-analysis
Source: Front Med (Lausanne). 2026 May 28;13:1808915. doi: 10.3389/fmed.2026.1808915 (PMC13253639; doi:10.3389/fmed.2026.1808915)
Supplement: Supplementary file 1 [file Table_1.docx]

Supplementary Table 1. Assessment of the risk of bias using the Joanna Briggs Institute critical appraisal checklist.

| **Study** | **Were the groups comparable other than the presence of disease in cases or the absence of disease in controls?** | **Were cases and controls matched appropriately?** | **Were the same criteria used for identification of cases and controls?** | **Was exposure measured in a standard, valid and reliable way?** | **Was exposure measured in the same way for cases and controls?** | **Were confounding factors identified?** | **Were strategies to deal with confounding factors stated?** | **Were outcomes assessed in a standard, valid and reliable way for cases and controls?** | **Was the exposure period of interest long enough to be meaningful?** | **Was appropriate statistical analysis used?** | **Risk of bias** |
| --- | --- | --- | --- | --- | --- | --- | --- | --- | --- | --- | --- |
| Chou SH et al. | NR | NR | Yes | Yes | Yes | No | No | Yes | Yes | Yes | Moderate |
| Endorf FW et al. | NR | NR | Yes | Yes | Yes | No | No | Yes | Yes | Yes | Moderate |
| Yang HT et al. | NR | NR | Yes | Yes | Yes | No | No | Yes | Yes | Yes | Moderate |
| Albright JM et al. | Yes | Yes | Yes | Yes | Yes | No | No | Yes | Yes | Yes | Low |
| Ligen L et al. | NR | NR | Yes | Yes | Yes | No | No | Yes | Yes | Yes | Moderate |
| Mosier MJ et al. | Yes | Yes | Yes | Yes | Yes | Yes | Yes | Yes | Yes | Yes | Low |
| Bai C et al. | NR | NR | Yes | Yes | Yes | No | No | Yes | Yes | Yes | Moderate |
| Spano S et al. | Yes | Yes | Yes | Yes | Yes | No | No | Yes | Yes | Yes | Low |
| Sutton T et al. | Yes | Yes | Yes | Yes | Yes | No | No | Yes | Yes | Yes | Low |
| Aung MT et al. | No | No | Yes | Yes | Yes | Yes | Yes | Yes | Yes | Yes | Low |
| Hu HC et al. | Yes | Yes | Yes | Yes | Yes | No | No | Yes | Yes | Yes | Low |
| Coulter JM et al. | Yes | Yes | Yes | Yes | Yes | Yes | Yes | Yes | Yes | Yes | Low |
| Dyson K et al. | No | No | Yes | Yes | Yes | Yes | Yes | Yes | Yes | Yes | Low |
| Walton NC et al. | No | No | Yes | Yes | Yes | Yes | Yes | Yes | Yes | Yes | Low |
| Coston DC et al. | Yes | No | Yes | Yes | Yes | Yes | Yes | Yes | Yes | Yes | Low |
